# The Complexity of Communication in Mammals: From Social and Emotional Mechanisms to Human Influence and Multimodal Applications

**DOI:** 10.3390/ani16020265

**Published:** 2026-01-15

**Authors:** Krzysztof Górski, Stanisław Kondracki, Katarzyna Kępka-Borkowska

**Affiliations:** 1 Institute of Animal Production and Fisheries, Faculty of Agricultural Sciences, University of Siedlce, Prusa 14, 08-110 Siedlce, Poland; stanislaw.kondracki@uws.edu.pl; 2 Department of Agriculture, Faculty of Technical Sciences, John Paul II University in Biała Podlaska, 95/97 Sidorska St., 21-500 Biała Podlaska, Poland; 3 Department of Genomics and Biodiversity, Institute of Genetics and Animal Biotechnology of the Polish Academy of Sciences, Postępu St. 36A, 05-552 Magdalenka, Poland; k.kepka@igbzpan.pl

**Keywords:** animal communication, multimodal signals, emotional communication, domestication, welfare

## Abstract

Communication in mammals constitutes a complex, multimodal system that integrates visual, acoustic, tactile, and chemical signals whose functions extend beyond simple information transfer to include the regulation of social relationships, coordination of behaviour, and expression of emotional states. This article examines the fundamental mechanisms of communication from biological, neuroethological, and behavioural perspectives, with particular emphasis on domesticated and farmed species. Analysis of sensory signals demonstrates that their perception and interpretation are closely linked to the physiology of sensory organs as well as to social experience and environmental context. In companion animals such as dogs and cats, domestication has significantly modified communicative repertoires ranging from the development of specialised facial musculature in dogs to adaptive diversification of vocalisations in cats. The neurobiological foundations of communication, including the activity of the amygdala, limbic structures, and mirror-neuron systems, provide evidence for homologous mechanisms of emotion recognition across species. The article also highlights the role of communication in shaping social structures and the influence of husbandry conditions on the behaviour of farm animals. In intensive production environments, acoustic, visual, and chemical signals are often shaped or distorted by crowding, noise, and chronic stress, with direct consequences for welfare. Furthermore, the growing importance of multimodal technologies such as Precision Livestock Farming (PLF) and Animal–Computer Interaction (ACI) is discussed, particularly their role in enabling objective monitoring of emotional states and behaviour and supporting individualised care. Overall, the analysis underscores that communication forms the foundation of social functioning in mammals, and that understanding this complexity is essential for ethology, animal welfare, training practices, and the design of modern technologies facilitating human–animal interaction.

## Simple Summary

Mammals communicate with one another using many different signals, such as facial expressions, body posture, sounds, touch, and smells. These signals help them express emotions, avoid conflicts, build relationships, and function safely in social groups. Animals that live closely with humans, such as dogs, cats, horses, goats, and farm animals, have adapted their natural ways of communicating to interact more effectively with people. Domestication has changed how these species use their voice, eyes, body, and behaviour, making some signals clearer and easier for humans to understand. At the same time, modern farming systems can limit the natural exchange of signals and make it harder to recognise signs of stress or discomfort. This review explains how mammals communicate across different senses, how emotions shape their behaviour, and how human influence has changed these processes. It also shows how new technologies, such as continuous video and sound monitoring, can help detect pain, stress, or illness early, improving the care and welfare of animals. Understanding how animals send and receive signals allows people to respond more accurately to their needs, strengthens relationships with domestic species, and creates safer, kinder, and more ethical environments.

## 1. Introduction

Communication is understood as the process of transmitting information from a sender to a receiver. Humans have developed highly complex systems and tools that enable rapid and precise exchange of diverse content, including over long distances and without direct physical interaction. A central component of this capacity is advanced verbal communication, grounded in spoken language. In the animal world, however, verbal communication occurs only in rudimentary forms and in a limited number of species. Among non-human animals, communication involves the transmission of signals between individuals of the same or different species. While some signals are produced intentionally to elicit specific behavioural responses, communication more broadly encompasses the transfer of information about an individual’s emotional or physiological state. Such information may be conveyed through behavioural, chemical, acoustic, or physiological cues, including hormonal signals such as cortisol or reproductive hormones, and does not necessarily result in an immediate or observable response from the receiver. Over the course of evolution, natural selection shaped emotions that enhance an organism’s ability to respond appropriately to environmental threats and opportunities [1]. In parallel, ecological and social pressures led to the emergence of species-specific repertoires of communicative signals, forming the characteristic “communication rituals” observed across taxa. Intraspecific communication is essential for regulating social distance, coordinating behaviour, and facilitating group decision-making. Animals exchange visual, acoustic, chemical, and body-movement signals, with the choice of modality influenced by environmental conditions and social context. The literature commonly distinguishes several primary channels of information transfer: tactile, chemical, acoustic, and visual communication [2]. Their functions are studied within the discipline of zoosemiotics, which aims to assess species’ capacities to develop symbolic signalling systems, interpret the meaning of signals in context, and evaluate the effectiveness of specific modalities under particular ecological conditions [3]. In interactions with humans, animals employ many of the same signals used in intraspecific communication. Dogs, for instance, closely observe human behaviour, are able to infer intentions, and can distinguish deliberate actions from accidental ones. Through their own signals primarily elements of body language they communicate emotions, needs, and motivational states [4,5]. Although animals possess a less elaborate communicative repertoire than humans, they rely on natural, evolutionarily shaped mechanisms of information exchange. Among canids, body language plays a central role in maintaining social hierarchies, preventing conflict, and initiating affiliative interactions such as play. In felids, vocal communication is comparatively more prominent: research indicates that acoustic signals carry greater functional weight than body language in both cat–cat communication and cat–human interactions [6,7].

In domesticated animals, interspecific communication, comprising interactions between heterospecific individuals, plays a particularly important role, especially in exchanges between animals and humans. Among the species that have developed highly specialised skills enabling flexible communication with humans, dogs are especially prominent [8]. They are also the species with the longest history of coexistence with humans, estimated in some sources to span approximately 30,000 years [9]. The ease with which dogs communicate with humans likely stems from the fact that they employ much of the same signalling repertoire in human-directed interactions as in intraspecific communication [10]. Importantly, many of these social-behavioural signals align closely with human social and emotional processing systems, facilitating mutual understanding, bonding, and cooperative interaction. In social species, accurate identification of another individual’s emotional state is essential, as emotions regulate the course of social behaviours and shape decision-making processes. In domesticated animals, emotional communication occurs both within species and in human–animal interactions. Research has demonstrated that dogs and horses can recognise the emotional content of human and conspecific signals based on single sensory modalities, including acoustic cues [11], facial expressions [12], and olfactory signals [13]. Evidence of interspecific communication has also been reported for other domesticated species [14,15].

This review integrates three complementary perspectives on mammalian communication: (1) the biological and ethological foundations of signalling across sensory modalities, (2) the role of emotions and neurobiological mechanisms in shaping communicative processes, and (3) the implications of communication for welfare assessment, training, and emerging technological applications, including Precision Livestock Farming and Animal–Computer Interaction. Rather than providing a purely species-by-species account, the review aims to identify shared principles underlying communication across domesticated and farmed mammals while acknowledging species-specific adaptations.

The aim of this article is to provide an integrated overview of current knowledge on mammalian communication—from its biological and neurobiological foundations, through the emotional and social mechanisms underlying information transfer, to the influence of domestication and human interaction on the evolution of communicative signals. This review synthesises findings from ethology, neurobiology, and applied behavioural sciences, with particular attention to practical applications in welfare assessment, training, and emerging technologies such as Animal–Computer Interaction (ACI) and Precision Livestock Farming (PLF).

The overarching objective is to present communication as a multidimensional system that integrates emotion, cognition, and social adaptation, and to highlight how understanding these mechanisms can improve human–animal relationships and the welfare of domesticated animal species.

## 2. Forms of Animal Communications

A wide variety of communicative forms exist in the animal kingdom, shaped both by the divergent evolutionary trajectories of species and by environmental changes associated with human activity. In domesticated species, the processes of domestication and subsequent selective breeding have further modified certain modes of communication. Animals rely on multiple senses and sensory modalities to transmit and receive signals [16]. These senses constitute the basis of the sensory apparatus that enables organisms to gather information about their surroundings. In most vertebrates, the primary sensory systems include vision, hearing, olfaction, taste, and touch. However, some groups have evolved additional modalities, such as electroreception in certain fish, magnetoreception in some bird species, echolocation in bats and cetaceans, or infrared detection in select reptiles [17]. Depending on the importance of each sense in the biology and behaviour of a given species, they have evolved into a single, characteristic form. The forms used by animals include visual communication, auditory communication, chemical communication, tactile communication, and body language [18,19].

### 2.1. The Role of Touch in Animal Communication

Tactile communication is a form of direct signal transmission and is typically preceded by other modalities, such as visual, acoustic, or olfactory cues. Because physical contact requires close spatial proximity, touch plays a particularly important role in social interactions in which individuals must approach one another. The most common forms of tactile communication include mutual rubbing, grooming behaviours, caregiving activities, and comfort-related actions such as licking, scratching, or leaning against one another. However, tactile signals may also function to communicate the need for increased distance or to establish behavioural boundaries between interacting individuals. Such signals can include biting, scratching, kicking, or forceful pushing and should be interpreted as context-dependent, dyadic communication reflecting immediate motivational states rather than as indicators of stable social rank or hierarchical structure. Tactile signals are detected by mechanoreceptors located within the epidermis and dermis [20]. In many species, vibrissae (specialised tactile hairs located primarily on the head) also contribute to fine mechanical perception and facilitate nuanced tactile exploration [21]. Tactile communication is especially widespread in species that form structured social groups [22]. Elephants, for example, use their trunk during social interactions to convey greetings, assess the reproductive or emotional state of partners, offer reassurance, and communicate information related to health [23,24]. Across many mammals, mutual grooming strengthens social bonds, particularly between mothers and offspring, and promotes post-conflict reconciliation and the reduction in social tension [22]. Touch also accompanies play and agonistic interactions, where it may serve as a signal initiating play, drawing attention, or contributing to the establishment of dominance. In such contexts, sequences of tactile behaviours may include nudging, gentle biting, or pushing [25]. Tactile contact is likewise an important component of communication between dogs and humans. Physical touch functions as an affiliative signal and exerts a calming effect on both the dog and its caregiver [26]. Human–dog relationships are strengthened through appropriately tailored physical contact—petting in preferred areas promotes bonding, reduces stress, and increases willingness to engage socially. Relaxed dogs show reduced reactivity and greater readiness to initiate or maintain interactions with humans [27].

Sensory systems play a pivotal role in the generation of sexual behaviour in animals. Such behaviours arise from the interaction between the organisms’ internal physiological state and external environmental cues that modulate arousal and readiness to mate [28]. Among the stimuli involved in communication between sexual partners, visual, acoustic, olfactory, and tactile cues are of primary importance, with their effectiveness determined by the sensitivity of the corresponding receptors and sensory organs [29]. Tactile stimuli become particularly significant at the moment of direct contact between males and females. Physical touch provides the male with information about the female’s receptivity, while simultaneously facilitating and enhancing her sexual responses. In pigs, for example, a sow in estrus responds to tactile stimulation from a boar by assuming a characteristic immobile, lordotic mating posture [30]. In the presence of a male, estrus behaviours in sows become more pronounced; females often adopt a rigid, arched-back posture that facilitates mounting and penetration. In some cases, contact with an inactive or inexperienced boar may elicit vigorous nudging or pushing by the female. In contrast, during the post-estrus phase, females exhibit aversion to tactile stimulation and reject mounting attempts [31]. In sheep, an estrus ewe may rub her neck and torso against a nearby ram, and tactile stimulation of her external genitalia by the male frequently induces urination, which functions as a combined chemical–behavioural signal [32]. Female cattle also actively participate in courtship interactions: they rub against the bull, sniff and lick his body, and, in the pre-estrus phase, become increasingly aroused and socially attentive. During this period, cows readily initiate contact with conspecifics, rubbing against them, licking them, and engaging in behaviours such as mounting, head pressing, and pushing [33,34].

### 2.2. Visual Communication

Visual communication relies on the use of vision to perceive signals generated by other individuals. Information is conveyed primarily through gestures, movements, and body postures. Visual signals consist of controlled motor patterns that incorporate morphological elements capable of attracting the receiver’s attention, such as movements of the ears, tail, muzzle, or other body parts [6]. External appearance, including colouration and body patterning, also plays an important role, serving as either social cues or deterrent signals. However, the effectiveness of this mode of communication is limited in species that are nocturnal or subterranean, where light availability is low [35,36,37]. Illumination is therefore a key factor influencing the efficiency of visual signals, under favourable lighting conditions, animals can employ more complex and subtle optical cues [38,39]. Despite this, visual communication can also be effective under dim-light conditions. Many nocturnal mammals possess dichromatic colour vision and contrasting achromatic patterns (black–white markings), enhancing signal discrimination, even under moonlight [36]. Some nocturnal species additionally exhibit ultraviolet sensitivity and biofluorescence, with their fur displaying fluorescent patterns upon exposure to UV light [40]. For instance, the distinctive facial markings of the plains vizcacha (*Lagostomus maximus*) may function as antipredator signals, similar to the contrasting facial rings observed in various lemur species [36,41].

The ability to perceive light, colours, and patterns depends on the sensory adaptations of each species, which is mirrored in the broad morphological diversity of mammalian colouration. For example, many rodent species exhibit black–white striping or other contrasting head markings, whereas in coatis the ringed tail serves as a visual signal facilitating group coordination during foraging or movement through dense vegetation [42]. Animals living in cohesive social groups commonly use variation in body posture and facial expressions, particularly those involving the head, to communicate emotional states and behavioural intentions [43,44]. Domestic sheep can discriminate characteristic structural and colour features of the head, not only in other sheep, but also in individuals of other species, including humans. Studies have demonstrated that sheep are capable of recognising human faces from photographs [45,46]. This ability has also been documented in horses [47] and dogs [48]. The capacity to identify unfamiliar faces from images is a trait shared by humans and certain primates [49,50], requiring sophisticated, holistic processing of visual stimuli [51,52]. Facial recognition of the same species has similarly been reported in cattle [53], horses [54], dogs [55], and goats [56].

Visual communication also plays an important role in sexual selection as well as in signalling age, condition, or social status. In pigs, sexual activity is shaped by a complex interaction between the animal’s physiological state and environmental stimuli, including visual cues generated by a potential mate [57,58,59]. In some primate species, colourful patches of skin and fur function as indicators of health, social rank, or reproductive readiness [60].

The use of colour in visual communication, particularly in signalling reproductive status and mediating social interactions, was described as early as Darwin’s classical writings on sexual selection [61]. Body colouration is widely employed in intraspecific communication, serving both social and sexual functions [62]. Distinctive and contrasting colours expressed by males and females may serve as indicators of genetic quality and reproductive potential, a phenomenon frequently observed in socially living primates [63,64]. For example, dark mane colouration in male lions is associated with superior physical condition and high testosterone levels, increasing male attractiveness to females [65]. In many primate species, males exhibit more intense colouration than females, facilitating the signalling of dominance and social status. Primates possess highly developed visual systems, and visual communication plays a central role in their daily interactions, with acuity and colour discrimination underpinning a wide range of social behaviours [66]. Colouration is often used to generate visual signals in body regions that are readily visible to conspecifics. In numerous primate species, facial and perineal colouration is particularly visible to individuals of the same species. The intensity, distribution, and patterning of these colours are strongly linked to male–male competition and status signalling [67,68]. In rhesus macaques (*Macaca mulatta*), for instance, males with more intense red facial colouration receive greater sexual acceptance from females than males with pale pink faces [69].

Animals also employ visual communication in antipredator contexts. Conspicuous colouration functions as a warning signal, indicating toxicity or the high cost of attack. Such signals consist of combinations of features including colour, contrast, symmetry, and body shape [70]. In terrestrial animals, aposematic displays often involve combinations of red, orange, and yellow [71]. Studies on the firebug (*Pyrrhocoris apterus*) have shown that predators attend more strongly to dominant colour than to specific pattern details [72]. Similarly, research on poison frogs of the genus *Epipedobates* demonstrates that their conspicuous colouration serves as an effective deterrent to potential predators [73].

### 2.3. Body Language

Animals convey essential information through body postures, facial expressions, ear and tail position, and eye expression. The ability to perceive and interpret the emotional signals of conspecifics is particularly important in species living in social groups [74]. Domestic animals have developed a rich repertoire of signals used in both intraspecific and interspecific communication, especially with humans. Dogs initiate play through the characteristic “play bow,” in which the forelimbs are extended forward and the hindquarters are raised, often accompanied by rapid tail wagging [75]. Dogs are widely regarded as exceptionally well adapted to human social environments; they exhibit high social tolerance and pronounced social attentiveness, enabling flexible adjustment to human actions and communicative cues [76]. This is particularly relevant in contemporary contexts in which dogs function as family members and partners in daily social interactions [77]. Additional play-related signals include dynamic hindquarter movements [78,79]. Rolling onto the back with the belly exposed, observed in both dogs and cats, may serve as an invitation to play rather than solely a signal of submission [80,81,82]. Social play fulfils an important communicative function and typically emerges under conditions of low stress. It is characterised by variability, flexibility, and a degree of unpredictability [83]. Despite its seemingly unstructured nature, play follows specific rules, and violations of these rules may lead to escalation, conflict, or even aggression [84]. In cats, the motivation to initiate play with a human frequently manifests as “stalking” behaviour performed nearby, accompanied by dilated pupils and forward-oriented ears, an expression indicating heightened arousal and readiness for interaction [82,85].

Body language is also used to communicate threat, submission, and attempts to regulate distance during social interactions, both toward other animals and toward humans.

In cats, such signalling involves approaching an intruder trespassing on occupied territory and maintaining prolonged, intense staring [86]. Preparatory cues for an attack include forward-directed vibrissae and elevated hindquarters, which visually “enlarge” the body. These threatening and aggressive behaviours function as communicative strategies aimed at deterring another individual or object and prompting withdrawal [87].

Dogs display a wide range of postures in which the configuration of the body reflects their emotional state, motivational context, and behavioural intentions [88]. An upright stance, a forward-extended chest, and piloerection along the neck may indicate that a dog is preparing to defend its territory or perceives a potential threat [10]. Additional threat-related signals include erect ears, tense facial musculature, and marked piloerection [89]. Dogs exhibiting threatening behaviour typically adopt a posture characterised by ears flattened or oriented forward, a raised tail, bristled neck hair, the head thrust forward, exposed teeth, tightened lips, rounded mouth corners, and wrinkling of the nasal region [90]. These behaviours should be interpreted as context-dependent communicative signals that may reflect fear, arousal, or defensive motivation, rather than exclusively as expressions of dominance or aggression.

In dogs, signalling confidence, vigilance, or perceived threat involves increasing apparent body size by straightening the posture, tensing the musculature, or extending to full height. Conversely, dogs can reduce their “perceived size” by lowering the body and tail and flattening the ears, thereby avoiding conflict in uncertain or challenging situations [10,91,92]. An upright posture is associated with dominance displays, whereas ear and lip position contributes to signalling aggression or submission [93]. A fearful dog adopts a submissive stance: the ears are laid back, direct eye contact is avoided, the tail is tucked, and the muzzle may appear wrinkled around the nostrils. The head is often lowered or turned aside, and the body assumes a crouched position with flexed limbs. Such a dog may also expose its abdomen while simultaneously covering the genital area with the tail, an unambiguous sign of submission [94]. In communication with humans, dogs additionally employ referential gestures, directing their gaze and orienting their body toward a specific object to elicit an appropriate response from the caregiver [4].

Facial expressions are another important communicative tool in both dogs and cats [4,95]. The configuration of the muzzle provides valuable cues about the animal’s emotional state and motivational stance. In dogs, retracted lips and forward-oriented ears indicate readiness to play. In contrast, a lowered head, ears pulled back, crouching, or extending the forelimbs are signs of withdrawal. Indicators of uncertainty or social discomfort include wide-open eyes directed toward the caregiver, slight ear elevation, and a closed mouth [96]. In cats, curiosity is expressed through intense staring at the human, forward-facing erect ears, and fully open, bright eyes, characterised by increased ocular openness and pupil visibility associated with heightened arousal and attentiveness [74].

Dogs and cats display characteristic facial expressions during states of depression or low mood. In dogs, such states are reflected in partially closed eyes, a sombre facial appearance, and noticeable relaxation of the facial musculature [97]. In cats, signs of sadness include a fixed, “vacant” gaze and reduced facial muscle tension. Conversely, content and relaxed individuals show overall bodily looseness; in cats the ears remain steady and motionless, whereas in dogs they may be slightly elevated [98,99]. Facial and head expressions therefore serve as valuable tools for assessing welfare in companion animals. Alterations in facial musculature or ear position may indicate the presence of pain or stress, a relationship documented in several species including cattle [100], horses [54], sheep [101], pigs [102,103], and cats [104]. Facial expressivity appears particularly developed in domesticated animals, likely due to their long evolutionary and social history with humans. A notable example is the ability of dogs to raise their eyebrows, producing a juvenile-like expression; this capability is absent in wolves, whose facial musculature does not allow such movements [105]. Facial expressions play a key role in nonverbal communication, enabling animals both to express their own emotions and to recognise the emotional states of others [106]. Body language and facial movements facilitate the expression of emotions such as happiness, sadness, surprise, disgust, anger, and fear. Owner-based studies have shown that behavioural indicators of fear in dogs include hiding or escape attempts, panting, yawning, lip-licking, avoidance of eye contact, a lowered body posture, ears pinned back, and a tail carried low or tucked between the legs [107]. Experimental studies using positive and negative stimuli demonstrate that dogs respond to positive cues by widening their eyes, orienting their gaze forward, and straightening and stabilising their ears [108].

The eyes are a central element of body language, functioning not only as sensory organs but also as active transmitters of social information. Among communicative signals, eye contact, particularly in dogs, stands out as one of the most influential and effective means of conveying information. Facial expression is a core component of emotional responses in social mammals, and dogs display a natural tendency to gaze at human faces and engage in eye contact in various contexts, including when requesting food or awaiting instructions [86]. Eyes are also used to transmit emotional states to other individuals. Staring at another animal or at a human may indicate a threat, whereas gaze aversion serves as a calming signal that reduces tension [96]. In relaxed situations, dogs’ eyes typically appear “soft”; in contrast, a hard, intense stare, often accompanied by a furrowed brow, signals high arousal or stress. Direct and prolonged eye contact with an unfamiliar human can be interpreted as a sign of aggression [96]. When confronted with an angry owner, dogs generally avert their gaze, displaying a de-escalatory response [78,108]. Eye-tracking studies show that dogs focus primarily on the eye region when processing the facial expressions of animals of the same species [109]. Cross-species emotion recognition is more challenging, as key emotional cues are often species-specific. Evidence suggests that domestication and prolonged coexistence with humans have shaped distinct visual signals in dogs that differ from those used in intraspecific communication. Domesticated dogs exhibit a spontaneous tendency to gaze at human faces and initiate eye contact [110]. Typically, such gaze behaviour functions as a request most often for food [111] or other forms of assistance. Thus, a gaze directed at humans operates as a solicitation signal [96], whereas in interactions between dogs the same behaviour would more commonly signal a threat or challenge [4]. This indicates that dogs employ eye contact differently with humans than with conspecifics. Gaze directly at humans has acquired a positive, affiliative meaning and facilitates dog–human interaction. Overall, these findings suggest that the domestication process has functionally modified gaze behaviour, adapting it for interspecific communication and aligning it more closely with human modes of social signalling [112].

Companion cats often occupy the same anthropogenic social niche as dogs, and their eyes play a key role in communication. Relaxed cats exhibit characteristic slow blinking, which serves as a signal of comfort and contentment. In contrast, frightened individuals display round, dilated pupils; pupil dilation also occurs during preparatory stages of attack or in self-defence situations [6,113]. Direct gazing in cats most commonly appears in contexts where they attempt to interpret human intentions or behaviour [114].

Ear movements and positioning also constitute essential communicative tools across many mammalian species. The ears of dogs and cats are highly mobile—each species possesses approximately 20 muscles controlling pinna orientation, enabling precise sound localisation as well as the expression of emotional states. In dogs, ear posture conveys substantial information about mood: slightly raised ears indicate security and contentment [4], erect ears enhance acoustic localisation [115], whereas ears held tightly back may signal intense fear [116,117]. Forward-directed ears typically denote interest. In cats, upright, twitching ears reflect heightened attentiveness to stimuli. Content and alert cats hold their ears upright and slightly forward. A cat anticipating a threat positions its ears straight back, whereas a frightened cat flattens its ears laterally against the head. Rapid ear flicking indicates the detection of a particularly salient sound [6,98,118]. A cat preparing to attack flattens its ears while slightly retracting the head; a fearful cat flattens its ears more drastically and withdraws the head further [74].

Body language and ear posture also play a crucial role in emotional expression in horses. Movements of the ears, eye reactions, and tension in the lips and nostrils provide rich information about an individual’s emotional state. Bored horses display loosely drooped ears positioned backwards and slightly apart, half-closed eyes, and relaxed, pendulous lips; yawning is common. They may also paw the ground or stamp, signalling impatience [119,120,121]. Vigilance is characterised by vertically oriented ears, wide-open eyes, flared nostrils, and an elevated head-and-neck posture [122,123]. Curious horses direct their ears toward the object of interest, stand squarely on all four limbs, and hold the tail raised or gently relaxed. Soft visual tracking and quiet snorting indicate positive engagement [124,125,126]. Content horses display softly positioned ears slightly angled backward and a gently lowered head [127,128]. Aroused horses exhibit upright ears, increased muscle tension, wide-open eyes, and an elevated tail, and such arousal may be positive (e.g., play, excitement) or sexual in nature. In this state, horses may run, buck, or engage in rolling behaviours [129]. Frightened horses hold their ears stiffly forward, tighten the lips, tense the body, widen the eyes such that the sclera becomes visible, and may produce loud vocalisations—whinnies, squeals, or snorts—signalling fear, threat, distress, or discomfort [130]. Indicators of aggression include rigidly positioned ears flattened backwards, a tense posture, and wide-open eyes with visible sclera. This configuration typically serves as a warning, though it may precede defensive actions such as kicking or biting. Ears held sharply backward signal uncertainty, irritation, overt threat, and readiness to attack [131,132,133]. Agonistic behaviours may be expressed by rearing on the hind legs, lifting the forequarters, and striking with the forelimbs. These actions can occur in aggressive contexts but are also frequently observed during play or heightened arousal and should therefore not be interpreted exclusively as indicators of dominance or fixed social order [134,135].

Across many mammalian species, the tail plays a central role in communication. Tail position and movement, in combination with facial expressions, form complex signal configurations that correspond to distinct emotional states. Owing to its length and mobility, the tail is a highly salient signalling structure, capable of conveying information over long distances and allowing for substantial variability in meaning [136,137]. The communicative use of the tail is best documented in domestic dogs. Tail posture and dynamics provide insight into the animal’s intentions and emotional state. A tail held high typically signals confidence, arousal, or the motivation to initiate interaction, such as during greeting or play [4,138]. A stiffly held tail may indicate anxiety or perceived threat [139], whereas a loosely hanging tail reflects relaxation and the absence of stress. A stiff, elevated tail often accompanies dominant behaviour. Conversely, a low, gently wagging tail is typical of subordinate individuals, while a tail tucked tightly between the hind limbs signals fear, uncertainty, or conflict de-escalation by reducing the animal’s apparent body size [140,141]. Dogs that are alert or highly stimulated wag their tails vigorously, often with wide-amplitude movements [10,93,142]. Tail base height has also been identified as an indicator of social status, a phenomenon first described in wolves [143] and later confirmed in domestic dogs [144]. In cats, the tail likewise constitutes a key component of body language. Rapid tail lashing generally signals arousal or irritation and often warns that the animal should not be approached [145,146]. A fully erect, piloerected tail indicates fear or aggression. A tail held straight and relaxed suggests a neutral state, whereas a vertically raised tail is an affiliative signal. Cafazzo and Natoli [147] termed this posture the “tail-up display”, characteristic of friendly social interactions among cats. Studies on urban feral cat colonies show that this signal occurs more frequently in females than in males and is more commonly expressed by lower-ranking individuals. A bristled tail held downward reflects unease or threat perception, whereas a tail tucked beneath the body indicates fear and submission [140]. A raised tail with a slightly lowered tip is often associated with excitement or positive anticipation. In horses, tail movements also serve as salient indicators of emotional state. Vigorous tail swishing against the flanks can indicate irritation or frustration, while a tail tightly clamped under the hindquarters suggests fear or uncertainty. In contrast, a tail held high is characteristic of horses that are joyful, excited, or highly aroused [148,149].

Across species, these postural and facial signals serve a common function of regulating social distance, signalling emotional state, and coordinating interactions, despite differences in morphology and ecological context.

### 2.4. Acoustic Communications in Animals

The ability to recognise conspecifics based on auditory signals has been demonstrated in dogs [91,150], horses [151], pigs [152], and cats [13]. Acoustic signals can travel over considerable distances, although their transmission is often disrupted by acoustic noise, which masks the original signal and impairs perception [153,154]. The auditory sense plays a critical role in the lives of both wild and domestic animals [155]. Because the hearing range of many species differs substantially from that of humans, animals may respond to sounds that are inaudible to their caretakers. Such reactions, triggered by stimuli beyond the human auditory threshold, can be sudden, intense, and difficult to predict, with important implications for animal safety and welfare [155,156]. The diversity of acoustic signals arises partly from anatomical variation in the vocal apparatus. Animals are capable of producing sounds through multiple mechanisms. For example, elephants communicate not only through vocalisations but also by generating seismic signals via ground impacts; their low-frequency rumbles facilitate individual recognition [157]. Animals use sounds across a broad frequency range, from infrasonic signals below 20 Hz to ultrasonic signals above 20 kHz. Certain rodent species communicate using infrasound below 10 Hz [158]. Ultrasonic signals, although less suitable for long-distance transmission, play a key role in the social communication of rodents, bats, and many cetaceans [159]. Bats, dolphins, and porpoises employ high-frequency sounds both for echolocation and for social communication. However, acoustic strategies among cetaceans are highly diverse. Some species, particularly large baleen whales, primarily use low-frequency sounds to communicate over long distances, whereas toothed whales, including dolphins, tend to rely on higher-frequency vocalisations such as whistles, chirps, and burst-pulsed sounds. An exception is the production of low-frequency narrow-band signals by some toothed whales in specific social contexts. In aquatic environments, sound propagates more rapidly and over longer distances than in air, further increasing the functional value of acoustic signalling in marine mammals [160]. The types of vocal signals produced also depend on the social system of a given species. Acoustic signals may serve as alarm cues, such as vocalisations emitted during territorial intrusions [161]. In gibbons and howler monkeys, loud calls are central to intergroup competition [162,163]. Many species use distinct calls to warn conspecifics of predators; for instance, marmots produce acoustically varied alarm whistles depending on the nature of the threat [164]. In reproductive contexts, males of numerous species employ acoustic displays as part of mate competition. During rutting season, male cervids emit powerful roars that signal physical condition and strength; individuals producing louder and more resonant vocalisations are preferred by females [165].

Dogs use eight principal types of vocal signals in intraspecific communication [166,167]. High-pitched barking is most commonly associated with submissiveness or juvenile behaviour, and in some contexts may indicate excitement, playfulness, or solicitation directed at a human handler or another dog. In contrast, low-pitched barking typically occurs in situations involving threat or territorial defence, particularly when a stranger approaches the dog’s territory [168,169]. Small dog breeds often exhibit intense, persistent barking, which functions as a warning signal directed toward larger animals [170,171,172].

Humans are able to distinguish among different types of barking and whining and to attribute distinct meanings to them, which facilitates the interpretation of canine intentions and enables appropriate behavioural responses [4]. Dogs use whining both as an attention-seeking signal directed at the caregiver and in submissive contexts, thereby communicating low social status. Whining may also indicate a need to go outside or enter a room, as well as discomfort or pain; in some dogs, it occurs without an identifiable external trigger [173]. Growling represents another vocal signal, most often preceding aggressive behaviour. A growling dog communicates the seriousness of the situation and typically demands an increase in distance. Although growling may function solely as a threat display, it is crucial to distinguish this form of signalling from cues that indicate a genuine risk of aggression [169]. Howling serves both as a long-distance communication signal between dogs and as an indicator of loneliness or anxiety. Vocal reactivity varies markedly between individuals [167,174] and is also influenced by breed. Some breeds (such as the Basenji, Chow Chow, or Shar Pei) show low vocal propensity, whereas others bark more frequently [172,175]. Barking primarily functions as a territorial signal, while whining is used mainly to elicit caregiving behaviours from conspecifics or humans [167,176].

Acoustic signals constitute important indicators of dogs’ emotional states and motivational processes. Analyses of vocalisations can discriminate between positive and negative affective states [177]. Research also suggests sex-related differences, with males exhibiting higher vocal activity than females [178]. Age additionally affects vocal behaviour: adult dogs, due to their established social position and caregiving roles, emit more acoustic signals (barks, growls, whines) than younger individuals [179,180].

Cats express both positive and negative emotional states through a diverse range of vocal signals. The most commonly produced vocalisation in domestic cats is the meow. Vocal activity is especially pronounced in kittens that have been properly socialised with humans from an early age. The feline vocal repertoire spans a wide spectrum of sounds, from soft purring to loud, intense calls associated with agonistic encounters or reproductive behaviours [181]. Domestic cats possess a more extensive and complex vocal repertoire than any other member of the order Carnivora and are substantially more vocal than their wild ancestor, the African wildcat (*Felis silvestris lybica*) [182]. In free-ranging cats, including those living in social groups, vocalisation is markedly reduced, their communication is far quieter compared to that of domestic cats [183]. Three primary contexts of feline vocal communication are traditionally distinguished: mother–offspring interactions, agonistic encounters (fighting, aggression, competition), and sexual behaviour [183,184]. To these one must add cat–human communication, which has emerged and diversified through the process of domestication. Research has demonstrated that cats can recognise their own name when spoken by humans [185]. Domesticated cats have been shaped not only by selective breeding but also by prolonged coexistence with humans, resulting in the development of specialised vocal strategies directed specifically toward people [186,187]. Meowing exemplifies this specialisation: it is rare in cat–cat interactions yet constitutes one of the most frequently used signals in cat–human communication. Meowing in its current form is believed to be a domestication-driven adaptation functioning primarily to attract human attention [188]. From an evolutionary perspective, it has been proposed that cats more effectively using meows to elicit caretaker responses would have received greater support and thus enjoyed higher survival rates [189].

Feline vocalisations can be broadly classified into three categories: tonal calls (meows), purr-like sounds, and loud defensive or aggressive signals such as hisses, spits, and screams [190]. In social contexts, short and gentle meows function as greeting signals or expressions of positive engagement with humans. Conversely, long, drawn-out meows often indicate frustration or dissatisfaction, particularly when the cat is attempting to prompt a specific human response. Purring is among the most frequently observed feline vocalisations and has a dual function: it typically signals comfort and contentment, but may also occur during stress, pain, or illness. Chattering a rapid, staccato sound usually emerges when a cat observes stimuli that strongly evoke predatory motivation, such as birds outside a window. During play, cats may emit short squeaks. Purring accompanied by forward-directed or laterally splayed vibrissae and tense facial muscles may signal irritation or serve as a warning of potential aggression. Loud hissing, spitting, screaming, and growling are characteristic of agonistic contexts and sexual encounters, occurring both as defensive responses to threats and as elements of competition or territorial defence [181,191]. Hissing functions primarily as a deterrent, whereas loud screams commonly accompany direct physical altercations or injuries. Growling in cats may serve both offensive and defensive roles, signalling readiness to fight or protect oneself. Demand meowing is typically loud and prolonged, with vocal intensity increasing progressively if the desired outcome is not achieved. Sex-related differences in feline vocalisations have also been documented. Males produce characteristic calls during courtship and copulation, whereas females vocalise more intensely during estrus and when interacting with their offspring [192,193].

Farm animals likewise use acoustic signals to communicate emotional states. In pigs, short grunts are associated with positive affect, whereas loud squeals often reflect pain or stress. Cattle express distress through low-frequency moos, while positive-emotion states such as excitement or anticipation are characterised by higher-frequency calls. Horses, although generally less vocal, emit soft whinnies as greeting or contact calls, whereas groans or grunts may indicate fatigue or boredom [156,194].

Despite species-specific sensory adaptations, the underlying communicative function of these signals is conserved across mammals, enabling the transmission of emotional and motivational information.

### 2.5. Chemical Communication

Chemical communication is based on the exchange of information encoded in odorant substances released by the organism. Olfactory cues influence the behaviour of both conspecifics and heterospecifics. The key advantages of chemical signals include their long persistence in the environment, the possibility of transmitting information regardless of the time of day, and their high efficacy even at very low concentrations. Animals use scent communication for species identification, sex and individual recognition, locating reproductive partners, deterring rivals, marking territories, and warning group members of potential threats [195,196,197]. Chemical cues are released via urine, faeces, and the secretions of numerous scent glands. In most mammals, these glands are located in the areas of the head, anogenital region, inguinal region, and paw pads. In many species, social encounters typically begin with sniffing of the anogenital area [198,199]. In felids, scent glands located around the head are particularly well studied, while in hoofed animals, chemical cues originating from the interdigital glands play a crucial role in social and territorial signalling [200,201].

Urine and faeces serve as additional sources of chemical signals used in communication [30,202,203]. Information encoded in faeces about the type of diet enables species or population recognition, and the composition of the gut microbiota may indicate an individual’s health status [204]. Many carnivores, such as the red fox, domestic cat, and dog, use faeces to mark territory and signal their presence [205]. During the mating season, urine provides information about an individual’s physiological status, with the scent of urine allowing discrimination of sex, health condition, and, in females, the phase of the reproductive cycle [206].

Information centres and deterrent signals can also be established through rubbing parts of the body against environmental objects, other individuals, or their own body. This behaviour is characteristic, for example, of bears and weasels [207,208]. Bats use chemical cues to recognise conspecifics, identify sex and colony members [209], and mark objects to deter rivals [210]. In some primate species, males deposit scent marks from their genital area during the breeding season, signalling reproductive condition and attracting females [211].

Chemical communication also plays a significant role in domesticated species. Dogs can discriminate individual humans by scent, and preferred sniffing regions on the body suggest that different anatomical areas emit distinct chemical cues [212,213]. It has been demonstrated that emotion-related odours, such as those associated with fear, joy, or anger elicit different behavioural responses in dogs [214]. Fear-related scents increase vigilance and anxiety, whereas odours associated with joy evoke affiliative behaviours, such as tail wagging or licking [215]. Depending on the emotional valence of the olfactory stimulus, dogs may also adjust the intensity of their interaction with humans, odours of familiar or bonded individuals increase interest and proximity seeking, whereas the scent of an unfamiliar person elicits greater caution [4].

Olfactory communication is equally important in cats. Through scent, cats mark territory, recognise individuals, and maintain social bonds. A key structure involved in chemical communication is the vomeronasal organ (Jacobson’s organ), located above the palate, which enables the detection and analysis of pheromones and other chemical cues [216]. Rubbing behaviour directed at objects, other animals, or humans serves both to exchange social information and to deposit the cat’s scent onto environmental elements. This enables, among other functions, the recognition of estrus females by males and the identification of shared territory [217].

Although species differ in their specific signalling repertoires, all mammals rely on four primary communication modalities: visual, acoustic, chemical, and tactile. Each provides distinct adaptive advantages and varying degrees of resistance to environmental disturbance. Table 1 summarises these modalities, outlining their functions, applications, and typical limitations.

## 3. Communication in Social Groups (Social Structures, Hierarchies, Networks)

Communication constitutes a fundamental mechanism underlying the organisation of social structures in mammals. While classical ethology often described social organisation in terms of linear dominance hierarchies, increasing evidence in domestic species such as dogs and horses supports more flexible, relationship-based social structures characterised by individual roles, social cohesion, and context-dependent agonistic interactions. Signals do not merely reflect existing relationships; they actively shape them by influencing group cohesion, the stability of social relationships, and the efficiency of information flow. Integrating classical research on animal communication with social network analysis (SNA) demonstrates that patterns of signal exchange reveal the direction, strength, and quality of social connections, and can predict consequences for individual welfare, social status, and reproductive success [218]. In most social species, relative social status and individual roles within groups are communicated using multiple modalities, including chemical, visual, acoustic, and postural signals. Such multimodal redundancy minimises the need for costly physical confrontations, replacing them with ritualised displays, threats, and appeasement behaviours. These “rules of engagement” stabilise group structure by reducing conflict escalation and lowering the risk of injury. From a neuroethological perspective, status-related signals engage specialised perceptual systems and modulate physiology, arousal, and behavioural responses in real time [219]. Individual recognition represents another key mechanism structuring social life. Variation in signal characteristics whether olfactory, vocal, facial, or behavioural supports the identification of partners, allies, and competitors, facilitating coordinated action and the maintenance of long-term social bonds.

This mechanism has been documented across numerous taxa and enables the precise “addressing” of signals within a social network [43]. Social network analysis provides quantitative metrics for assessing individual communication roles, such as degree, betweenness centrality, and closeness centrality. These metrics link local interactions to emergent properties of the entire group. Reviews indicate that individuals with high centrality function as “information hubs”, accelerating the spread of alarm signals, behavioural innovations, or feeding preferences [220]. SNA also enables cross-species comparisons and integration of long-term behavioural datasets with modern tracking technologies. In many mammals, decisions regarding movement, foraging, or structural changes in fission–fusion societies emerge from local interaction rules embedded within the network’s topology. Both modelling studies and empirical data demonstrate transitions from centralised structures dominated by a single leader to decentralised systems in which decision-making is distributed among group members. Communication patterns determining who sees, hears, or follows whom influence the speed, efficiency, and accuracy of collective decisions [221].

Modern research techniques such as telemetry, biologgers, accelerometry, and computer-vision systems allow the integration of spatial, behavioural, and multimodal communication data. At the same time, generative network models enable the testing of hypotheses about the emergence and dynamics of social structures in natural and anthropogenically altered environments. A major challenge remains the full integration of communication research (signal modality, content, and context) with network topology analyses, which is essential for understanding the mechanisms governing social life in mammals [218]. Only through a combined analysis of “who interacts with whom and how often” (SNA) and “what, how, and in what context is communicated” (signal analysis) can we capture the processes through which communication creates, regulates, and transforms animal societies. In domestic species, particularly dogs and horses, such communication patterns often reflect relationship-based social organisation and context-dependent interactions rather than rigid, linear hierarchies. Taken together, these examples illustrate that social communication in mammals relies on flexible, relationship-based interactions rather than uniform, species-specific signalling systems.

### 3.1. The Role of Emotions and the Neurobiology of Communication

Animal communication relies not only on the exchange of sensory stimuli but also on the ability to recognise, interpret, and mirror the emotional states of conspecifics. Emotions constitute the neurobiological foundation of social behaviour, modulating attention, motivation, and responses to communicative signals. In social mammals, the capacity to perceive the emotions of group members and, in domesticated species, also human emotions is crucial for maintaining social bonds and behavioural synchrony [222,223,224].

Neuroimaging studies demonstrate that mammalian brains exhibit homologous activation patterns when processing emotional signals. In both humans and dogs, similar areas of the temporal cortex and amygdala are activated in response to vocalisations expressing positive or negative affect [150]. Electroencephalography (EEG) and functional magnetic resonance imaging (fMRI) analyses show that dogs display heightened activity in limbic structures and the prefrontal cortex when exposed to emotionally charged sounds, whether canine or human [225]. This ability likely evolved during domestication and represents a co-evolutionary perceptual adaptation between humans and dogs [226,227]. Comparable neural correlates have been documented in cats, which can discriminate human emotional states based on vocal intonation and facial expressions [74]. These mechanisms align with the concept of emotional mirroring, which involves mirror-neuron networks in the frontal and parietal cortices [228]. Emotion recognition requires the integration of multisensory information—including visual, auditory, olfactory, and tactile cues. Dogs and horses can match facial expressions with vocal tone, detecting emotional congruence across modalities [229]. Similar cross-modal integration has been shown in primates, where activity in the superior temporal sulcus increases in response to emotionally congruent audiovisual stimuli [230]. In dogs, this process involves coordinated activation of visual, auditory, and limbic regions within an occipito-temporal cross-modal network identified using fMRI [231]. From an ethological perspective, emotions serve as regulators of social interactions. Positive affect promotes affiliation, play, and bonding, whereas negative emotions (e.g., fear, anger) signal threat and initiate avoidance or defensive responses. In many species (dogs, primates, and dolphins) emotion perception enables flexible behavioural adjustments to the social context [223].

Emotions also modulate facial expression, vocalisation, and posture, influencing how signals are interpreted by receivers [97]. Emotion recognition is closely linked to neurohormonal processes, particularly oxytocin and vasopressin, which mediate social bonding. In dogs, mutual gaze with a human caregiver increases oxytocin levels in both partners [226]. This “oxytocin–gaze loop” provides a biological basis for interspecies empathy and emotional communication. Emotional expression has clear neurophysiological correlates. Facial movements, ear posture, and eye configuration reflect emotional arousal and welfare state. Changes in facial muscle tension correlate with activity in premotor cortex and basal ganglia, a phenomenon demonstrated in primates [232], pigs [103], and cats [233]. The development of grimace scales has enabled quantitative assessment of emotional expression, particularly in the context of pain and welfare [103].

The neurobiology of animal communication thus reveals that emotions are not merely responses to stimuli but integral components of social signalling. Domestication has enhanced certain species’ (e.g., dogs’) capacity to perceive human emotions and has reshaped neural circuits involved in processing social information. Emotions therefore form a shared “interspecies language” a system of signals that enables cooperation, empathy, and mutual understanding between animals and humans.

### 3.2. The Influence of Domestication and Human Interaction on Communicative Signals

Domestication alters not only the morphology and neurophysiology of signalling systems but also the functions and use of the signals themselves. These phenomena are captured within the concept of the domestication syndrome, which links selection for tameness and human-directed cooperation with developmental modifications in neural crest cell populations. Such changes have secondary effects on facial musculature, ear and tail morphology, pigmentation patterns, and other structures that serve as carriers of visual and acoustic signals [234]. In dogs, one of the most thoroughly documented outcomes of domestication is the evolution of specialised facial musculature that facilitates communication with humans. Of particular importance is the *levator anguli oculi medialis* muscle (“inner brow raiser”), which is absent in wolves. Activation of this muscle elevates the inner portion of the eyebrow, drawing human attention and enhancing the emotional salience of the signal. This represents a morphological adaptation of the visual signalling channel to interspecies communication [227]. Moreover, mutual gazing between dogs and humans triggers a bidirectional increase in oxytocin levels, forming a positive feedback mechanism, the so-called oxytocin–gaze loop, which strengthens social bonding and promotes cooperative tendencies. This mechanism does not occur in wolves and is interpreted as a product of coevolution between canine signalling strategies and human perceptual biases [226]. In cats, components of the vocal repertoire have undergone functional shifts toward human-directed communication. A classical example is the solicitation purr, a purr containing a high-frequency element that effectively elicits caregiving responses from humans. Recent research also demonstrates context-dependent variation in meow prosody, supporting the idea of adaptive refinement of the acoustic channel in cat–human interactions [182,189]. Domesticated ungulates likewise show human-oriented communicative behaviours. Goats, for instance, modulate gaze and employ alternating looks in the “unsolvable task” paradigm, with their behaviour depending on human attentional cues such as body orientation and face direction [235]. This indicates that selection for cooperation and close cohabitation with humans has favoured the emergence of referential signals and socially mediated “tool use” in interspecies communication [235].

Not all outcomes of domestication, however, enhance communicative efficiency. Selective breeding that drastically alters skull shape and facial configuration can reduce the range of facial expressions and diminish emotional signalling precision relative to ancestral forms. Comparative studies of canine facial expression reveal that some breeds exhibit a constrained ability to produce specific affective displays, a noteworthy counterpoint to the assumption that domestication universally improves communication [236].

Domestication and human influence therefore operate along two parallel pathways: (1) they selectively amplify signals that effectively engage human perception and emotion (e.g., canine eye expressions, feline vocal prosody, referential gaze in goats), while (2) secondary morphological modifications may constrain the expressive repertoire and reduce signalling precision. Understanding both of these vectors is essential for interpreting behaviour, assessing welfare, and designing evidence-based training practices for domesticated species [182,226,227].

The process of domestication has profoundly modified the forms and functions of communicative signals in dogs, producing a clear departure from patterns characteristic of wolves. As a result, dogs display heightened sensitivity to human social cues and have developed a set of behaviours specifically adapted for interspecies communication. Table 2 summarises key differences in wolf–dog–human communication, illustrating how selective pressures associated with cohabitation with humans have generated unique communicative mechanisms in dogs, distinct from those found in their wild ancestors and from typical human signalling systems.

### 3.3. Communication in Farm Animals

Despite domestication, farm animals often inhabit environments that diverge substantially from their natural ecological and social conditions. Production systems and facility infrastructure may restrict the expression of the full natural communicative repertoire. Nevertheless, these animals continuously perceive external stimuli and respond to them accordingly. Noise associated with high stocking density, mechanical equipment, and human traffic within housing facilities can induce hyperarousal, sleep disturbances, distress, and ultimately impair psychological health [237]. Vocalisations produced under intensive housing conditions do not always reflect species-typical communication; instead, they frequently express emotional states. Accurate interpretation of these signals provides caregivers and producers with valuable information regarding animal welfare and may guide interventions to reduce stress [156].

Cattle exhibit a strong herding instinct; isolation from conspecifics triggers stress and fear, whereas excessive stocking density can promote somatic disease. Cows employ a diverse set of postural and acoustic signals [238]. Affiliative interactions, such as allogrooming and bodily rubbing occur most frequently between individuals of comparable hierarchical status. Four major categories of cattle vocalisations have been described, each associated with distinct emotional states; modulation of vocal features facilitates functions including social coordination and alarm calling during pasture movement [184,239]. Estrus in cows is marked by characteristic behavioural patterns, including increased locomotion, mounting behaviour, and soliciting contact with other animals. Chronic stress arising from inadequate nutrition or restricted social contact may lead to abnormal behaviours such as reduced lying time, compulsive cross-sucking, licking of pen structures, or excessive self-grooming [33,240].

In pigs, acoustic and postural cues constitute the dominant communication channels. Vocalisations occur during foraging, play, and most prominently in threatening situations. The vocal repertoire includes grunts, squeals, and screams, which vary in frequency and amplitude. Intense screaming is most commonly observed in piglets competing for access to teats, whereas sow grunts function as contact calls that summon piglets during nursing [239]. Piglets also grunt during play, signalling positive arousal. Tail posture is an important welfare indicator: tail rigid extension in European breeds often denotes pain or illness. Changes in body posture play critical roles in hierarchy formation and can signal aggression, submission, or attempts to withdraw [156,241].

Sheep are highly gregarious and exhibit strong flocking behaviour, including synchronised feeding, resting, and movement. Leadership within the group is typically assumed by the oldest ewe, whereas among rams the dominant individual is usually the strongest. Agonistic behaviours include butting, pushing, and visual standoffs. Acoustic cues are central to sheep communication: low-frequency bleats from ewes facilitate mother–lamb contact, and rams produce characteristic rutting calls during the breeding season [242]. Sheep are sensitive to disruptions in visual contact, which may result in behavioural and neuroendocrine dysregulation [239]. Their sensory capabilities include sophisticated olfactory perception and enhanced auditory sensitivity compared to humans [243].

In goats, social hierarchy correlates strongly with age, with older females typically occupying higher positions. Tactile communication plays a significant role—individuals often rest close together, lick each other, engage in nibbling of the coat, and participate in muzzle-to-body contact. Vibrato-like bleating indicates anxiety, while loud, repetitive bleats function as contact calls to offspring. Sneezing serves as an alarm signal. Snorting and tongue-flicking are common in sexual contexts and accompany behaviours such as biting, butting, and tail grasping [244]. During the breeding season, strong odours arise from the activity of peri-anal glands combined with increased urination frequency. Environmental deficiencies—poor housing, inadequate milking systems, low-quality feed—can promote behavioural abnormalities including aggression and butting [239,245,246].

Alpaca herds are organised around a social hierarchy; males engage in dominance contests involving body elevation, spitting, pushing, and biting of limbs and the neck. Such confrontations may result in significant injuries, including testicular damage leading to reproductive exclusion. A male that abandons the contest typically crouches and retreats with its head lowered [247]. The principal acoustic signal is a soft humming vocalisation, whereas neigh-like calls, warning snorts, and high-pitched squeals indicate a threat [239,248]. Spitting functions as a mechanism of social control within the group. Disruptions of communication may result from isolation, inadequate housing conditions, or premature weaning [249].

Donkeys communicate using acoustic, visual, olfactory, and postural signals. Olfactory recognition plays a pivotal role in mother–foal bonding. Ear posture is an important emotional indicator: interested donkeys hold the ears upright with eyes widely opened, whereas relaxed individuals display lateral ear positioning, a lowered lower lip, and a horizontally aligned neck. Agitated donkeys flatten the ears, lower the neck, and deliver sidekicks [239,250]. Vocalisations occur more frequently in males than in jennies or geldings. Impaired communication is most commonly associated with isolation, improper nutrition, inadequate husbandry conditions, or premature weaning [251].

Chickens possess a highly complex acoustic communication system. Short, rapid calls warn of aerial or terrestrial predators. Broody hens produce numerous vocalisations of varying pitch and frequency that guide chick behaviour [239,252,253].

Horses form hierarchical herd structures, and visual perception constitutes their primary mode of communication. They possess a panoramic visual field of approximately 350°, with binocular vision of around 65° [254]. Acoustic communication enables long-distance contact, expression of arousal, and mother–foal interactions. Tactile communication strengthens social bonds; the lips, tongue, and muzzle are particularly sensitive. Olfactory cues facilitate territorial marking and play an essential role in social relationships [17].

Vocalisations constitute a fundamental component of communication in livestock species, serving to signal emotional states, physiological needs, and socially relevant information. In production environments, where direct behavioural observation may be limited, acoustic analysis provides valuable insights into animal welfare and stress. Table 3 synthesises the principal types of vocalisations observed across major livestock species and interprets their behavioural and emotional significance. This overview highlights the diagnostic potential of acoustic signals for assessing animal condition and for improving husbandry practices.

## 4. Multimodality and Practical Applications (Welfare, Training, ACI/PLF)

Animal communication is inherently multimodal: visual, acoustic, tactile, and chemical signals are combined into integrated packages designed to increase detectability, enhance robustness against noise, and improve interpretive clarity. Theoretical frameworks emphasise that these modalities may (1) complement one another by providing redundancy, (2) carry distinct informational content, thereby offering complementarity, or (3) be flexibly selected depending on the receiver’s attentional state, the social context, and environmental conditions. Methodological challenges remain, including the distinction between genuinely multimodal signals and multi-element but single-channel displays, as well as the development of unified measurement standards allowing meaningful cross-species comparisons [255,256]. Empirical work provides clear evidence of multimodal signalling; for instance, greeting rituals of African savannah elephants integrate coordinated visual and tactile gestures with vocal elements (rumbles, trumpets). Modality choice depends strongly on the receiver’s attentional orientation: when the receiver is visually attentive, visual signals dominate; in its absence, tactile and acoustic cues become more frequent. This phenomenon represents a compelling example of audience design in mammalian communication [257]. Domesticated species with high social sensitivity, such as dogs and horses, exhibit cross-modal integration during the recognition of human emotions. Consistency in valence between facial expressions and vocal prosody enhances interpretive accuracy, suggesting that integrative mechanisms promote signal reliability under conditions of sensory noise [258].

In practice, animal training when based on clear, multimodal cues and appropriate reinforcement can function as a form of environmental enrichment. Training facilitates the use of other enrichment elements, modifies social interactions, and broadens the animal’s repertoire of adaptive behaviours, thereby contributing meaningfully to welfare. A prerequisite for effective training is the design of communicative cues that are cross-modally consistent and tailored to the species’ perceptual capacities [259].

The growing relevance of Animal–Computer Interaction (ACI; in agriculture also HACI–human/animal–computer interaction) and Precision Livestock Farming (PLF) stems from their ability to integrate multiple data streams–vision, sound, accelerometry, environmental sensors–to enable continuous monitoring of behaviour and welfare. Reviews indicate that coupling ACI/PLF with artificial intelligence can support personalised husbandry and increase animal agency through interactive systems that facilitate access to resources (e.g., self-initiated positive stimuli, microclimate selection). However, challenges remain in relation to scalability, data ethics, and alignment with practical farm constraints [260]. In cattle-monitoring systems, combining visual gait analysis with accelerometry, feeding-time metrics, activity patterns, or thermal imaging improves the detection of lameness and allows intervention before pain intensifies or production losses occur. Despite rapid progress, translational barriers include calibration across farms and algorithmic stability under variable environmental conditions. In poultry production, multisensor systems evaluate movement patterns, changes in vocalisation, and environmental parameters, enabling early detection of stress and health issues [261]. Smart-farming technologies can meaningfully improve welfare only when the monitored indicators are designed around the needs of the animals rather than solely around production efficiency. Essential prerequisites include field validation of welfare-relevant indicators, transparency of decision algorithms, and the use of collected data to generate tangible improvements in husbandry conditions (e.g., microclimate optimisation, noise reduction, behavioural interventions). Only such an approach allows multimodal ACI/PLF systems to be implemented ethically and in a manner aligned with the biological and behavioural needs of animals [262].

### Practical Implication

Understanding the mechanisms of animal communication has not only theoretical value but also clear practical relevance for improving welfare, enhancing training effectiveness, and advancing technologies that support human–animal relationships. Recognising that signals are inherently multimodal and emotionally grounded enables the design of more empathetic and effective interactions that align with animals’ natural perceptual capacities as well as with the constraints imposed by domestication and environmental conditions. Research on multimodal communication demonstrates that animals much like humans integrate visual, acoustic, tactile, and chemical cues into coherent social representations, thereby increasing signal recognition accuracy and reducing the likelihood of misinterpretation [255,263]. In practical terms, this means that training and everyday handling should rely on consistent cross-modal cues, such as the simultaneous use of gestures, vocal tone, and eye contact. Training based on clear, predictable, and concordant communicative signals reduces stress, shortens learning time, and strengthens the human–animal bond [259]. Applied ethology increasingly emphasises that training should be regarded not only as a utilitarian tool but also as a form of environmental enrichment. Numerous studies show that training grounded in positive reinforcement and transparent contingencies lowers physiological indicators of stress (e.g., cortisol, heart rate variability), enhances exploratory and affiliative behaviours, and improves the quality of cooperation between animals and humans [97,264]. Communication thus functions simultaneously as a medium for behavioural guidance and as a mechanism for establishing trust and emotional well-being.

In parallel, technologies enabling objective monitoring of emotions and health are developing rapidly. Precision Livestock Farming (PLF) systems and Animal–Computer Interaction (ACI) interfaces integrate data from video, acoustic analyses, thermography, accelerometry, and environmental sensors to create digital models of behaviour and welfare [260,265]. Analyses of vocalisations, facial expressions, or movement patterns allow early detection of pain, stress, metabolic disorders, or lameness [261,266]. When designed from the animal’s perspective, these systems can support genuinely personalised care, identifying individual needs and preferences rather than assessing animals solely through the lens of production metrics [267].

A central challenge concerns the ethical orientation of technological development. Neethirajan et al. [260] emphasises that the future of sustainable animal production depends on combining artificial intelligence with principles of ethology and the neurobiology of emotions. Monitoring systems should be engineered to respond to indicators of stress or discomfort in ways that mirror the actions of an empathetic caregiver. The Human–Animal–Computer Interaction (HACI) framework similarly proposes that animals become active users of technology, capable of initiating contact, selecting preferred stimuli, or modulating environmental features [268]. This perspective reframes technology as a facilitator of animal agency rather than an instrument of control. At the level of individual relationships, research on the oxytocin–gaze loop in dogs [226] and on emotional mimicry and referential behaviours [227] demonstrates that interspecies communication relies on mutual emotional attunement. In training, therapy, and caregiving contexts, humans can leverage these co-evolved mechanisms to interpret signals more accurately, reduce problematic behaviours, and enhance animals’ sense of safety.

Integrating ethological research, the neurobiology of emotion, and multimodal signal analysis leads to a model of communication characterised not as a one-way transmission but as a bidirectional dialogue. Practically, this implies the need to design environments, training methods, and technological systems that respect animals’ natural communicative repertoires, meet their emotional and cognitive needs, and support their welfare.

To synthesise the practical consequences of multimodal communication in mammals, Table 4 outlines the key domains in which signal integration and advances in ACI/PLF technologies influence welfare, training, and management systems. The summary highlights both the biological foundations of communication and their applications in husbandry, behavioural therapy, and human–animal interaction contexts.

## 5. Conclusions

Communication in mammals is a complex process that integrates biological, emotional, and social mechanisms. Although its primary role is information transfer, communication also regulates social relationships, maintains group cohesion, and enables behavioural adaptation. Most species employ multiple communicative channels: visual, acoustic, tactile, and chemical in a multimodal manner, which increases signal robustness and interpretative clarity.

Neurobiological evidence shows that emotional communication relies on homologous brain structures across mammals, and the ability to perceive emotional cues is evolutionarily conserved. In domesticated species, long-term coevolution with humans has further refined these skills, shaping both the form and function of communicative signals. Domestication has enhanced sensitivity to human social cues but, in some cases, selective breeding has constrained expressive capacities.

A systemic perspective integrating social context, emotional processes, environmental conditions, and evolutionary history is essential for understanding animal communication. Such an approach has practical implications for training, welfare assessment, and environmental enrichment. Emerging technologies, including Precision Livestock Farming (PLF) and Animal–Computer Interaction (ACI), enable objective, real-time monitoring of behaviour, stress, and health by combining multimodal data streams.

Ultimately, communication forms the foundation of mammalian social life. Recognising and respecting these processes is key to ethical human–animal interactions. Future research should continue to integrate ethology, neurobiology, and digital technologies to support and enhance communication in ways that improve both animal welfare and the quality of human–animal relationships.

## Figures and Tables

**Table 1:** Communication modalities in mammals.

Modality	Signal Sources	Example Species	Functions	Advantages	Limitations
Visual	Facial expression, posture, ears, tail	Dog, cat, horse	Emotions, status, threat signalling	High speed	Light-dependent
Acoustic	Vocalisations	Dog, cat, cattle, pig, birds	Distance signalling, alarm calls, mother–offspring communication	High speed	Prone to noise interference
Chemical	Pheromones, urine, scent glands	Cat, dog, ungulates	Territory marking, reproduction	High persistence	Slow transmission
Tactile	Sniffing, licking, rubbing, grooming	Cattle, goats, cats	Bonding, appeasement	High reliability	Requires close proximity

**Table 2:** Comparative overview of communicative traits in wolves, dogs, and humans, with emphasis on domestication-related changes.

Feature	Wolf	Dog	Effect of Domestication
Facial expression (mimicry)	Limited facial expressivity; reduced mobility of brow musculature	Presence of specialised musculature enabling eyebrow lifting (e.g., *levator anguli oculi medialis*)	Enhanced visibility and emotional salience of eye-region cues; increased effectiveness of human-directed signalling
Vocal behaviour	Low frequency of barking; vocalisations used mainly in long-distance communication and agonistic contexts	High propensity to bark; broad functional range including alarm, solicitation, attention-seeking, and affiliative contexts	Functional expansion of barking and diversification of vocal repertoire toward human-directed communication
Gaze behaviour	Direct eye contact avoided; prolonged gaze interpreted as threat	Spontaneous gaze seeking; use of gaze alternation and referential looking in social situations	Coevolution with human perceptual preferences; establishment of the oxytocin–gaze loop facilitating bonding and cooperation
Chemical communication (pheromones, scent marking)	Central modality for territoriality, group cohesion and reproductive status	Reduced reliance on scent marking in many contexts; greater use of visual and acoustic channels in interactions with humans	Decreased functional importance of chemical signalling in human-associated environments; behavioural shift toward multimodal social communication

**Table 3:** Vocalisations of livestock species and their behavioural/emotional significance.

Species	Vocalisation Type	Behavioural/Emotional Significance
Cattle	Low-frequency mooing	Stress, separation distress
Pig	Squeal	Pain, stress, acute distress
Sheep	Bleating	Ewe–lamb contact call, solicitation
Horse	Neigh/whinny	Long-distance contact, greeting, arousal/excitement

**Table 4:** Multimodal communication in mammals: Practical implications for welfare, training, and ACI/PLF technologies.

Domain	Key Insights	Practical Relevance
Multimodality of signals	Animals integrate visual, acoustic, tactile, and chemical cues; cross-modal congruence enhances perceptual accuracy	Designing coherent, multi-channel cues during training; reducing stress through clear communication
Emotionality of signals	Communicative signals reflect emotional valence and arousal; neural mechanisms are homologous across many mammals	Welfare assessment based on facial expression, vocalisation, and movement; use of training methods that promote positive emotion
Human–animal communication	Domesticated species (dogs, cats, horses, goats) recognise human facial expressions, prosody, and referential gestures	Improved cooperation and safety during interactions; development of training protocols based on clear human cues
Training as environmental enrichment	Positive reinforcement and clear signals reduce cortisol and improve social relationships	Implementation of welfare-focused training programmes in zoos, laboratories, shelters, and farms
PLF technologies	Multisensor systems (video, audio, thermography, accelerometry) detect stress, pain, lameness, and behavioural changes	Early diagnosis of disease; automated environmental adjustments (ventilation, lighting, feeding); personalised management
Animal–Computer Interaction (ACI/HACI)	Animals can actively initiate interaction with technology; interactive systems enhance agency	Increased control over the environment; self-initiated rewarding stimuli; ethical design of technological interfaces
Limitations and challenges	Issues of scalability, interpretive errors, data quality, and variation across farms	Need for validated welfare indicators; transparent algorithms; integration of ethological and engineering knowledge

## Data Availability

No new data were created or analyzed in this study. Data sharing is not applicable to this article.
